# Task-dependent neural representations of salient events in dynamic auditory scenes

**DOI:** 10.3389/fnins.2014.00203

**Published:** 2014-07-21

**Authors:** Lan Shuai, Mounya Elhilali

**Affiliations:** Laboratory of Computational Audio Perception, Department of Electrical and Computer Engineering, Center for Speech and Language Processing, Johns Hopkins UniversityBaltimore, MD, USA

**Keywords:** attention, auditory scene, stead-state response, bottom-up, top-down

## Abstract

Selecting pertinent events in the cacophony of sounds that impinge on our ears every day is regulated by the acoustic salience of sounds in the scene as well as their behavioral relevance as dictated by top-down task-dependent demands. The current study aims to explore the neural signature of both facets of attention, as well as their possible interactions in the context of auditory scenes. Using a paradigm with dynamic auditory streams with occasional salient events, we recorded neurophysiological responses of human listeners using EEG while manipulating the subjects' attentional state as well as the presence or absence of a competing auditory stream. Our results showed that salient events caused an increase in the auditory steady-state response (ASSR) irrespective of attentional state or complexity of the scene. Such increase supplemented ASSR increases due to task-driven attention. Salient events also evoked a strong N1 peak in the ERP response when listeners were attending to the target sound stream, accompanied by an MMN-like component in some cases and changes in the P1 and P300 components under all listening conditions. Overall, bottom-up attention induced by a salient change in the auditory stream appears to mostly modulate the amplitude of the steady-state response and certain event-related potentials to salient sound events; though this modulation is affected by top-down attentional processes and the prominence of these events in the auditory scene as well.

## Introduction

Selecting relevant information from an auditory scene is guided either by the salience of the acoustic events (bottom-up driven) or by behavioral goals (top-down driven). While bottom-up and top-down attentional mechanisms engage different neural circuits of sensory processing and cognitive control (Buschman and Miller, 2007), their net effect on the neural representation of acoustic events appear to share many similarities (Katsuki and Constantinidis, 2014). Indeed, both forms of bottom-up and top-down attentional mechanisms have been reported to give rise to enhanced or better tuned responses to a relevant stimulus relative to the background, both at the single neuron level as well as the population level reflected in network analyses, event-related potentials and interactions across brain areas (Näätänen, 1992; Katsuki and Constantinidis, 2014). Specifically, saliency-driven attentional mechanisms are greatly reflected in the stimulus representation at the level of sensory cortex as well as the propagation of information to frontal areas, all while modulating key markers of the neural response as reflected in event-related components such as Mismatch Negativity (MMN) (Näätänen and Michie, 1979; Näätänen et al., 2007; Kim, 2014). Such changes are often accompanied by further modulation due to task-demands and guided by top-down attention which also affects sensory and frontal cortical networks (Picton and Hillyard, 1974b; Luck et al., 2000; Fritz et al., 2007a; Kiefer, 2007; Elhilali et al., 2009; Müller et al., 2009; Xiang et al., 2010). These effects are not unique to the auditory modality. They have been widely reported in vision as well (Buschman and Miller, 2007; Zhaoping, 2008; Andersen et al., 2012; Zhang et al., 2012), with stronger suggestions of independence between bottom-up and top-down attention despite some of their shared measureable effects (Pinto et al., 2013).

The role of attention in guiding processing of auditory scenes parsing is further regulated by the complexity of the scene itself, as well as the dynamic nature of the sound streams competing for a listener's attention. There is strong evidence suggesting that some of the neural mechanisms engaged in parsing complex acoustic scenes are in fact independent of top-down attention (Bregman, 1990; Sussman and Steinschneider, 2001). These attention-free processes are mostly a reflection of the acoustic properties and statistical nature of the stimulus which can bias its organization into mental representations perceived as segregated streams or salient events in the scene. In other words, parsing an acoustic scene is partly dictated by the physical nature of the acoustic cues present and the statistical evolution of these cues over time; which not only define the perceptual boundaries of the different auditory objects in the soundscape, but also direct the brain's computational resources to the relevant events in the scene based on their conspicuity and sensory relevance. These in turn can facilitate the process of top-down attention (rather than only vice versa).

In the current study, we try to tease apart the neural signature of bottom-up and top-down components of attention in a series of experiments, by focusing on the representation of salient events under or away from the spotlight of top-down attention. We embedded a salient change in a dynamic sound stream, and controlled listeners' attention to or away from this stream; both in presence or absence of a second competing sound stream; while measuring their neurophysiological responses using Electroencephalography (EEG). Key to our paradigm is that salient events are not defined by a simple form of deviance detection. Salience is defined here in a statistical sense that salient events diverge from the feature distribution of the dynamic auditory stream. This definition is chosen to clearly dissociate it from commonly used deviance-detection paradigms or odd-ball designs which rely on a standard sound event that is either physically repeated a number of times or with at least one of its acoustic properties of interest held fixed and repeated to establish a standard reference. In contrast, the paradigm presented here relies on a notion of standard that is defined only in a statistical sense, in that the feature of interest in the signal never repeats, but rather is drawn from a constrained statistical distribution. The implication of this design is that our brain is collecting or estimating these statistics as the signal evolves over time; and it infers that any changes from the underlying distribution as a violation of the statistical structure of the signal that would be deemed salient. Our working hypothesis is that the presence of a salient sound token as part of an auditory stream would trigger neural changes that reflect both its deviance from the existing object as well as reflect its salience as dictated by its prominence in presence or absence of competing streams. Such salient neural representation would be modulated by the attentional state of listeners, though parts of its neural signature would be independent of it. This hypothesis implies a degree of independence in the observed neural changes between bottom-up and top-down attention, whereby variations in the neural response due to salient changes in the acoustic structure of the scene could be observed and comparable in size independently of whether listeners were attending to the sound stream or not.

Our analysis focused on the auditory steady-state response (ASSR) as well as event-related potentials (ERPs) that were associated with the target sound stream which contained salient tokens. The ASSR analysis provides us a window into entrainment effects caused by the repeating nature of the stimulus and the degree to which they are modulated by both attentional state and complexity of the scene. These results complement the analysis based on event-related potentials; since the ASSR analysis is a frequency-based decomposition of a sequence of neural responses assessing their phase-locked nature, while the ERP analysis is a time-locked profile of the neural response that ignores the phase and pattern-locked changes over time though would indirectly influence the ASSR since a stronger stimulus-locked steady-state response would reflect a stronger match in the repeating pattern and hence a stronger alignment between responses to individual stimulus events. While there is an indirect association between these two components, they are not directly mapped from one to the other (Capilla et al., 2011; Zhang et al., 2013) and provide complementary views into the effect of stimulus and attentional manipulations on the neural response.

## Materials and methods

### Stimuli

**Figure 1:**
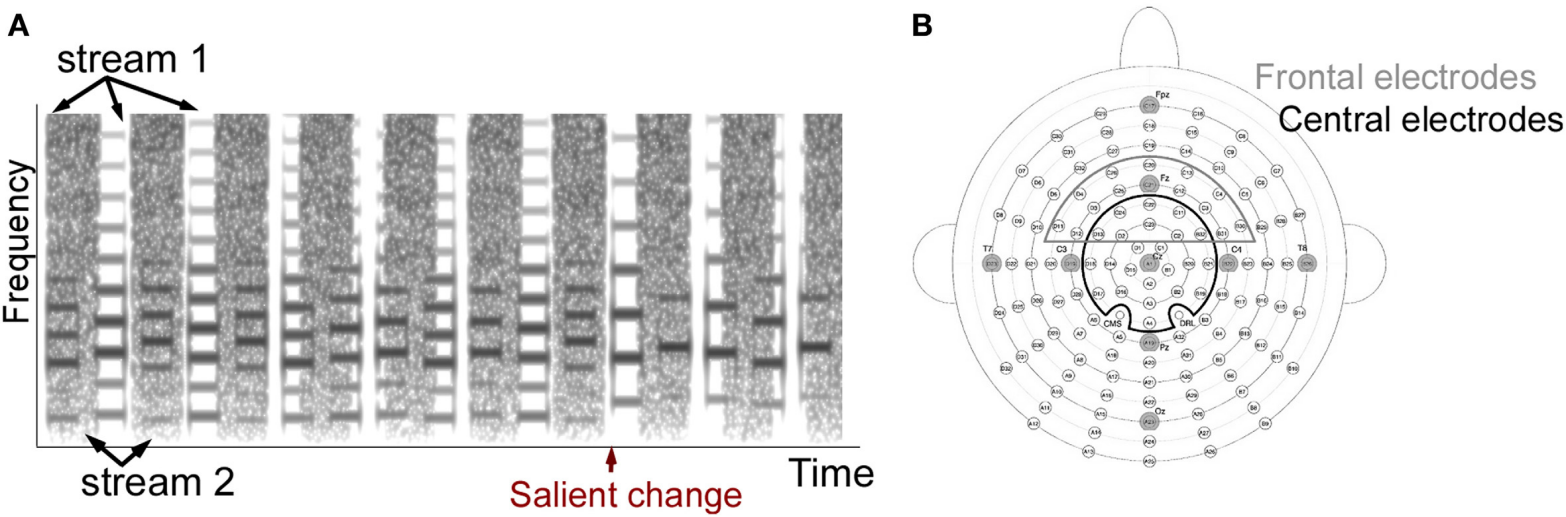
**(A)** Example stimulus spectrogram used in the two-stream experiments. The spectrogram of one sample trial from the two-stream experiment is shown. The stimulus consists of a musical notes stream and a sequence of noise bursts. The one-stream experiment did not include the noise stream. **(B)** Electrode positions in the 128-channel Biosemi system. Cz and 23 electrodes around Cz were included as the Cz electrode group, and Fz and 21 electrodes were included as the Fz electrode group. Original layout is available at: http://www.biosemi.com/pics/cap_128_layout_medium.jpg.

A total of three experiments were performed where both stimuli and attentional state of listeners were manipulated. One experiment included single-stream stimuli, and the other two included dual-stream stimuli (Figure 1A). The single-stream stimuli consisted of a sequence of concatenated synthesized musical notes. The notes were synthesized from spectral templates of three instruments: trumpet, saxophone and clarinet from the RWC instrument database (Goto et al., 2003; Goto, 2004). Each note in the sequence was defined by four features: intensity, duration, pitch and timbre (spectral template). Each of the four features was drawn from a discrete distribution consisting of 12 levels. These 12 levels were separated into three groups. Level 1–4 belonged to the first group, 5–8 belonged to the second group, and 9–12 belonged to the third group. By separating notes this way, feature changes within each group were smaller while changes between groups were larger on average. Detailed parameters of each feature dimension and each group are listed in Table 1.

**Table 1:** **Stimulus parameter space for stream 1 stimuli**.

**SPECTRAL TEMPLATE (TRUMPET: T; SAXOPHONE: S; CLARINET: C)**				
Group 1 [Level 1–4]	1^*^T	4.5/5.5^*^T + 1/5.5^*^S	3.5/5.5^*^T + 2/5.5^*^S	2.5/5.5^*^T + 3/5.5^*^S
Group 2 [L 5–8]	1.5/5.5^*^T+4/5.5^*^S	0.5/5.5^*^T+5/5.5^*^S	0.5/5.5^*^C+5/5.5^*^S	1.5/5.5^*^C+4/5.5^*^S
Group 3 [L 9–12]	2.5/5.5^*^C+3/5.5^*^S	3.5/5.5^*^C+2/5.5^*^S	4.5/5.5^*^C+1/5.5^*^S	1^*^C
**PITCH**				
Group 1 [L 1–4]	196 Hz (G3)	208 Hz (G3#)	220 Hz (A3)	233 Hz (A3#)
Group 2 [L 5–8]	247 Hz (B3)	262 Hz (C4)	277 Hz (C4#)	294 Hz (D4)
Group 3 [L 9–12]	311 Hz (D4#)	330 Hz (E4)	349 Hz (F4)	370 Hz (F4#)
**DURATION**				
Group 1 [L 1–4]	40 ms	50 ms	60 ms	70 ms
Group 2 [L 5–8]	80 ms	90 ms	100 ms	110 ms
Group 3 [L 9–12]	120 ms	130 ms	140 ms	150 ms
**INTENSITY**				
Group 1 [L 1–4]	60 dB	61.25 dB	62.5 dB	63.75 dB
Group 2 [L 5–8]	65 dB	66.25 dB	67.5 dB	68.75 dB
Group 3 [L 9–12]	70 dB	71.25 dB	72.5 dB	73.75 dB

Each experimental trial started with an auditory stream consisting of a sequence of notes drawn from the same feature group. Salient notes would then appear in the stream at a pseudo-random moment in the sequence, in which the sound tokens were drawn from a different feature group. Control trials did not contain any salient notes, but consisted of only a stream whose notes varied within one feature group. There were a total of 400 experimental trials and 80 control trials. The order of trials was randomized across subjects. In each trial, only one dimension of the features was changing both during the auditory stream and salient events segment. For example, a given trial would be made of music notes with varying pitch values (e.g., group1 pitches: G3–A3#) but fixed timbre, intensity and duration features. The salient change would share the same timbre, intensity and duration but introduce a higher pitch drawn from group 2 or group 3 (see Table 1). Each note within one group had the same probability to appear, and no adjacent notes were the same. The SOA (stimulus onset asynchrony) was set to 213 ms, hence the tempo of the entire sequence was fixed at 4.7 Hz. Each note in the sequence included a 10 ms cosine ramp at onset and offset. The auditory stream contained 6, 12, 18, 24, or 30 notes; each repeated 80 experimental trials. Within each 80 trials, 20 trials had one of the four feature dimension changes. Ten of 20 trials had greater level jump (group 1 to 3 or vice versa) and 10 had smaller level jump (e.g., group 1 to 2). The salient change segment was composed of 12 notes. The control trials consisted of 16 trials of 18, 24, 30, 36, or 42 notes. The length of control trials matched the whole duration of experimental trials.

The dual-stream stimuli consisted of two streams playing simultaneously (Figure 1A). The first stream was similar to the note sequence described above. Simultaneously, a second stream of modulated noise tokens was played (Figure 1A). The second stream consisted of a sequence of flat spectrum white noise tokens that were sinusoidally-modulated at 3 cycles/octave (Chi et al., 1999) with either modulation depth 0 (flat) or 0.35 (modulated). A trial consisted of either flat noise tokens with a modulated deviant; or modulated noise tokens with a flat deviant. The deviant always appeared in one of the last 3 tokens in the sequence; with each of the 3 last positions being a deviant with equal probability. Control trials did not contain any deviant tokens. Each instance of noise was generated separately in order to avoid memorizing the temporal sequence of the noise. A modulation depth of 0.35 was chosen based on a pilot study indicating that this modulation depth was around the threshold of detection for average listeners, as modulation of 0.4 was easily detectable and 0.3 was hardly detectable. This value was slightly higher than previous reports of modulation detection thresholds in white noise (Chi et al., 1999) and could be due to the existence of the competing sound stream which increases the difficulty of the task.

The noise streams also comprised 480 trials; half consisting of flat noise standards and half of modulated noise standards. Fifty percent of all trials were controls with no deviant (half of them with flat noise standards, and half with modulated noise standards). Each noise token was 230 ms long with 10 ms onset and offset cosine ramps; and the SOA was set to 382 ms (corresponding to a tempo of about 2.6 Hz). The entire noise stream was composed of 11, 14, 17, 21, or 24 sound tokens to match the length of the music notes stream. The noise streams were randomly added to the music notes stream to form the dual-sound stream after setting the intensity of both sound streams to the same value over the full trial length.

The experiments were organized in three conditions: (1) attend to a salient change in the music sound stream (one-stream attend); (2) attend to change in the music sound stream while a noise sound stream is simultaneously playing in the background (two-stream attend); and (3) ignore music sound stream while attending to the noise sound stream (two-stream ignore).

### Participants and procedure

Thirty-six healthy volunteers between 18 and 35 years old (mean age: 21.81; std: 3.94) with no history of hearing problems or neurological disorders according to self-report participated in the experiments with informed consent. Due to the length of experimental conditions, subjects were randomly assigned to one of the three experimental conditions described above (12 subjects in each experiment), and compensated for their participation. All experimental procedures were approved by the Homewood Institutional Review Board (HIRB). Subjects sat in a comfortable chair inside a dim lighted sound-dampened chamber. Ambient noise inside sound booth was around 30 dB SPL tested by a sound level meter. A computer screen was placed 1.5 meters in front of participants. Participants wore a pair of ER-3A insert earphones during the experiment. Sound pressure level from the earphones was adjusted to a range between 60 and 80 dB SPL measured by a sound level meter.

EEG recording was done by using a 128-channel Biosemi Active Two system (Biosemi Inc., The Netherlands) with a sampling frequency at 2048 Hz (Figure 1B). Offsets of each channel were kept under 40 dB examined after preparation. Online filters were set from 0 to 417 Hz following the default values. Left and right mastoid electrodes were also recorded and used as averaged mastoid references during offline processing. The nose channel was recorded serving as a reference to check for existence of a Mismatch Negativity (MMN) component. Eye artifacts were monitored by recording four EOG channels. Two were placed below left and right eyes and two were put outside of the left and right outer canthus.

The whole experiment lasted about 2.5 h including about 45 min of preparation. Participants were asked to detect the presence of a salient change in each trial and respond after the trial ends by pressing “yes” or “no” buttons on a response box using their left and right index fingers. For half of the participants in each experiment, “yes” button was on the left and “no” answer was on the right, and for the other half it was the opposite. The position of the buttons was randomly assigned to participants. There were 10 practice trials to let participants get familiar with the task. If hit rate was under 60%, participants were given a second 10-trial practice session. A d-prime measure was used to assess the accuracy of detecting a deviant.

### Data processing

EEG signals were down sampled to 512 Hz and lowpass filtered at 104 Hz using a Decimator software provided by Biosemi, and then further preprocessed using FieldTrip (Oostenveld et al., 2011), MATLAB (MATLAB Release 2013a, the MathWorks, Inc., Natick, Massachusetts, United States), and EEGLab (Delorme and Makeig, 2004). Each data segment included 1065 ms (duration of 5 music notes) immediately before or after the deviant point in the sound stream. Data were re-referenced to the averaged mastoid electrodes as the reference, and a de-mean was applied to each segment to align the average at zero. A denoising procedure was applied by first bandpassing all signals between 0.7 and 30 Hz. Bad channels were marked by an experienced experimenter and flagged as channels with unusual high frequency noise or large drifts. They were replaced by the arithmetic mean of the surrounding channels. No more than two bad channels were replaced for each participant. Eye artifacts were removed by excluding correspondent one or two eye-blink components and one eye-movement component after ICA (Independent Component Analysis) decomposition. Finally, a second 0.7–30 Hz bandpass filter was applied to smooth over minor distortions occasionally introduced by ICA reconstruction. In order to ensure that trial onset effects were not contaminating our analysis, we only analyzed 320 trials out of 400 experimental trials which contained more than one second of the auditory stream.

After data preprocessing, ASSR and ERP responses were analyzed separately. ASSR was derived by first concatenating all trials in each condition for each participant over the 5 notes duration before and after a salient change. A Fourier transform was then applied to obtain the spectral distribution. The amplitude at 4.7 Hz (1/213 ms) was calculated to capture the stimulus-locked steady-state neural response (**Figure 3A**). The ASSR amplitude was confirmed by verifying that a peak at 4.7 Hz was greater than the averaged energy at surrounding frequencies across all participants and conditions (2–6 Hz excluding 4.7 Hz served as baseline). The ASSR analysis was derived from 24 central electrodes (Cz group) around the vertex as shown in Figure 1B (including electrodes A1 (Cz), A2, A3, A4, B1, B2, B19, B20, B21, B32, C1, C2, C11, C22, C23, C24, D1, D2, D13, D14, D15, D16, D17, D18 in the layout of 128-channel Biosemi headcap). The lateralization of ASSR was calculated by averaging ASSR peaks in the 56 left and 56 right electrodes excluding the midline electrodes.

Event-related potentials (ERPs) were analyzed by averaging all trials over the duration of two music notes (i.e., 426 ms) immediately before or after the deviant point with baseline correction using 100 ms prior to the segments. This analysis was called 1-note analysis in the results section in order to highlight that the focus is on the early and late components relative to the onset of 1-note. Because of the closeness in timing between successive music notes in the stimulus (SOA 213 ms), the analysis of the late ERP component of a given note was contaminated by onset effects of the following note. The ERP waveform at 0–213 ms was therefore subtracted from the ERP waveform at 213–426 ms in order to analyze any putative late components, including a P300 component. This waveform correction for analysis of the P300 component was repeated using other forms of correction (no correction, or by subtracting onset effects of a standard note instead of deviant one). Though different forms of correction resulted in slightly different shapes of the corrected waveform and peak latencies, all methods of a putative P300 analysis resulted in the same statistical results in terms of significant changes in the P300 component as a function of top-down attention and acoustic saliency.

The analysis based on 1-note required averaging across fewer trials (320 trials) and was repeated using an average of three consecutive music notes (3-notes) with more data (960 trials). In the 3–note analysis, data from 3 notes was averaged then baseline-corrected using 100 ms prior to the onset of the averaged segments. In order to avoid overlapping between the auditory stream and salient change, data starting from the 2nd last music note before the salient event served as standard and the 1st music note after the salient event served as deviant for the 1-note analysis. The data starting from 2nd, 3rd, and 4th last music notes before the salient event served as standard and the 1st, 2nd, and 3rd music notes after the salient event served as deviant for the 3-note analysis. The enlarged N1 was prominently visible only in the 1-note analysis and concealed changes in the positive P1 peak and the presence of a frontal MMN-like negativity which were only revealed with the 3 note analysis. The late P300 component was consistent across the 1-note and 3-note analysis.

ERP components were defined as follows: (i) An early P1 positivity was defined as a positive peak in standard and deviant ERP curves over the range 30–100 ms from the central Cz electrode groups. We defined the peak latency of the component as the average peak latency across all participants. Once a positive peak was identified, the amplitude over a window of ±15 ms around this peak was averaged and checked for statistical significance relative to the variance in the data. The peak latency for the P1 component was found to be 64 ms for all 3 experiments (1-stream attend, 2-stream attend and 2-stream ignore). The central topography of the P1 peak was confirmed by full head topography to verify that the maximal positivity is indeed localized in the central electrodes. (ii) An early negativity (N1) was analyzed similarly as a significant negative trough in both the standard and deviant ERP curves over the time range 100–213 ms in the Cz electrode group, with analysis window ±15 ms around lowest negativity. The peak latency for the N1 component was found to be at 156 ms for 1-stream attend, 176 ms for 2-stream attend and 166 ms for 2-stream ignore. (iii) An MMN-like component was defined as a significant negativity over 100–213 ms in the difference curve between standard and deviant in the frontal electrodes from the Fz electrode group as shown in Figure 1B (electrodes B30, B31, B32, C2, C3, C4, C11, C12, C13, C20, C21, C22, C23, C24, C25, C26, D2, D3, D4, D11, D12, D13). The frontal distribution of the MMN-like component was confirmed by full head topography with either the averaged mastoid reference or the nose reference. The peak latency for this component was found to be at 141 ms for 1-stream attend, 197 ms for 2-stream attend and 182 ms for 2-stream ignore. (iv) A late P300 positivity was defined as a significant positive peak in the difference curve over the time window 300–426 ms in the central Cz electrodes (corrected by ERP curves of the following note at 0–213 ms, as explained earlier). The peak latency for the P300 component was found to be at 360 ms for 1-stream attend, 410 ms for 2-stream attend and 410 ms for 2-stream ignore.

The complex nature of the variables manipulated in the current study required an across-experiment strategy without a full factorial design. Therefore, we conducted a number of statistical tests to alleviate concerns of comparisons across pools of subjects in different experiments. A One-Way ANOVA was performed on the behavioral results in order to detect any differences of performance across experiments. For the analysis of neural responses using ASSR and ERPs, we first conducted pairwise *T*-tests to investigate the effects of the salient event. Since bottom-up attention was one of the most important factors in our study, we reported the effect of salient change in each group as well with Bonforroni correction on multiple comparisons. Apart from the effect of salient change, we also examined the influences of (1) top-down attention or (2) auditory scene complexity on: (a) the auditory stream before the salient change by a univariate analysis on ASSR or MANOVA on ERPs and (b) the increase introduced by the salient change by looking at the interaction between saliency and attention or scene complexity in the Two-Way ANOVA. We also compared the lateralization of auditory stream and the salient change by using pairwise *T*-tests.

## Results

**Figure 2:**
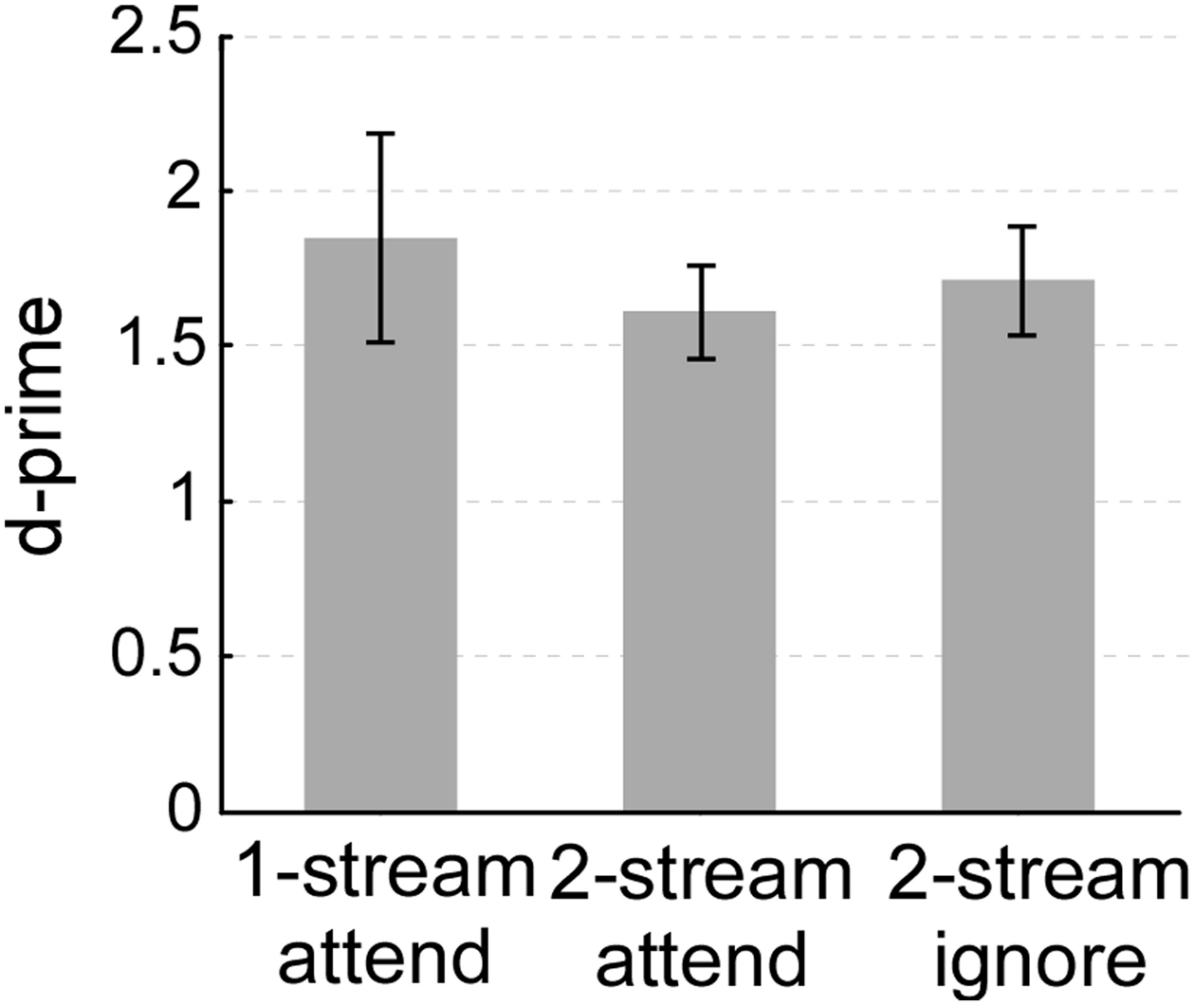
**Behavioral performance (d-prime) in the one-stream attend, two-stream attend or two-stream ignore experiments.** Error bars denote one standard error.

Though the complexity of the listening environment differed across experiments (presence of one or two streams), the ability of listeners to indicate the presence of a salient change or deviant in the attended stream was comparable. All three experiments yielded an average d-prime performance between 1.5 and 2 (Figure 2). A One-Way ANOVA confirmed that there were no statistical differences in performance between all 3 experiments (ANOVA between the three experiments: [*F*_(2, 33)_ = 0.259, *p* = 0.733, η^2^_*p*_ = 0.015]; between one-stream attend and two-stream attend [*t*_(22)_ = 0.646, *p* = 0.528]; or between two-stream attend and two-stream ignore [*t*_(22)_ = −0.443, *p* = 0.662)]. All three tasks were far from ceiling, hence engaging participants' selective attentional processes to a similar degree.

**Figure 3:**
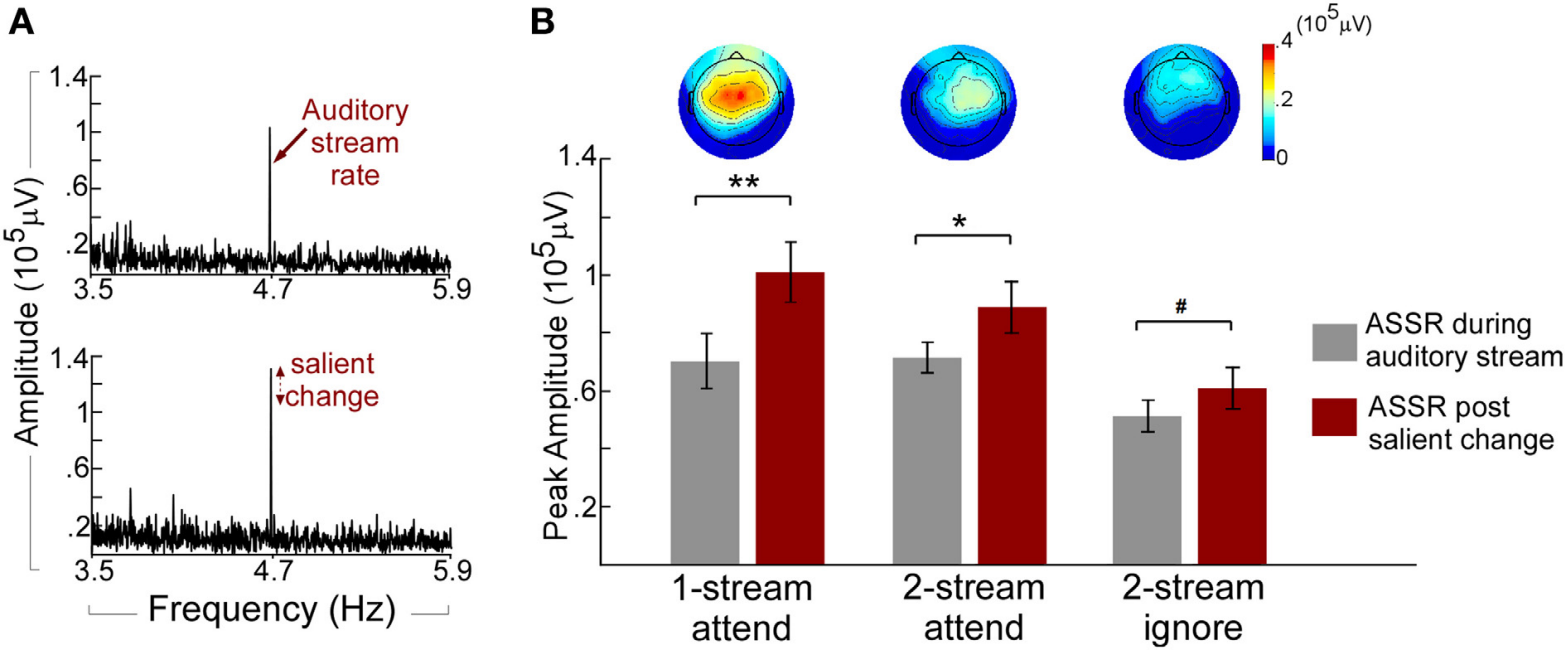
**(A)** Example ASSR peak during the auditory stream and post salient change. The spectrum of the neural response for one participant in the one-stream attend experiment is obtained by concatenating 320 trials at the Cz electrode. The Top panel shows the spectrum obtained during the auditory stream segment of the trial (prior to any change) and shows a clear peak at 4.7 Hz entrained to the tempo of the music notes. The Bottom panel depicts further enhancement in the 4.7-Hz peak after a salient change is introduced in the stimulus **(B)** ASSR amplitudes of the auditory stream and salient change and topographies of the ASSR increase in the three experiments. Each bar depicts the population average of ASSR peak before (left) and after (right) a salient change in the auditory stream in the Cz electrode group. Error bars denote one standard error. The topographies show the scalp distribution of the ASSR increase (change from before to after salient change). All topographies are shown to the same amplitude range. ^**^p < 0.01; ^*^p < 0.05; ^#^*p* < 0.1.

The neural responses reflecting the presence of a repeating pattern in the musical notes stream (whether attended or not) show a strong 4.7 Hz component in the neural signal of individual listeners (Figure 3A). Figure 3A (top panel) depicts the Fourier transform of the neural response for one participant in the one-stream attend task during the “standard” portion of the music auditory stream revealing a clear peak at 4.7 Hz entrained to the tempo of the music notes. This peak is further enhanced (Figure 3A, bottom panel) once a salient change occurs in the musical note stream. It is important to highlight that while its tempo is fixed, the musical notes stream used in this study is not deterministic by nature. It is characterized by small ongoing fluctuations along pitch, timbre, loudness, or duration. Despite this dynamic nature, the presence of a salient-enough change in the stream caused further enhancement to the phase-locked ASSR component. Figure 3B shows an analysis of the population response, and reveals that the enhancement was statistically significant irrespective of attentional state (attend or ignore experiments), or complexity of the auditory scene (one stream or two streams); with a central to frontocentral topography across experiments. A pairwise *T*-test on all participants revealed a significant increase of ASSR during the salient event [*t*_(35)_ = 5.239, *p* < 0.001; Bonferroni adjustment for the following three comparisons *p* = 0.017: one-stream attend: *t*_(11)_ = 4.123, *p* < 0.002; two-stream attend: *t*_(11)_ = 3.015, *p* < 0.012; and marginal significant increase in the two-stream ignore: *t*_(11)_ = 2.186, *p* = 0.051]. This strength in phase-locked responses is akin of previously reported effects of neural representations of salient, rare, unexpected events, particularly at the level of auditory cortex (Gutschalk et al., 2005; Bidet-Caulet et al., 2007; Chait et al., 2012).

**Figure 4:**
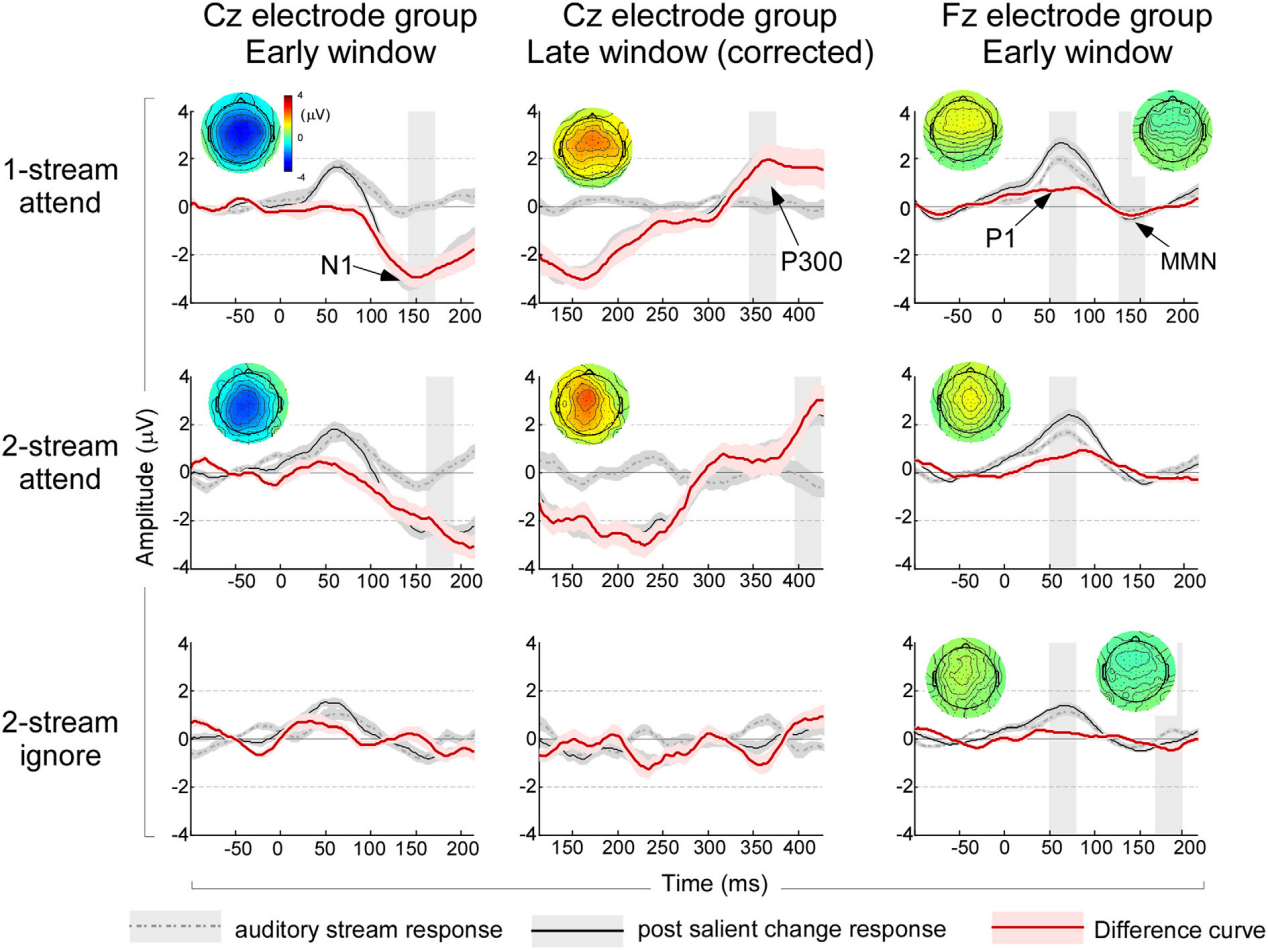
**Left column:** ERP waveforms and topographies in the early window immediately after onset of the music note from the Cz electrode group. The waveforms are based on analysis of 1-note before (dashed gray line) and 1-note after (solid black line) a salient change, in addition to a difference curve (solid red line). The shaded curves indicate a standard error around the mean waveform. A shaded time interval indicates a 30 ms window where a statistically significant N1 peak is observed, along with the full head scalp topography of the N1 peak in the same analysis window. **Middle column:** ERP waveforms and topographies in the late window after note onset corrected for onset effects of the following note from the Cz electrode group. The analysis follows the same structure shown in the left column and time intervals and topographies indicate significant P300 peaks in a 30 ms time window. **Right column:** ERP waveforms and topographies immediately after onset from analysis of 3-notes before and after a salient change from the Fz electrode group. The first significant time interval indicates timing of the P1 peak (analyzed from the Cz electrode group—not shown); while the second time interval around 160 ms indicates the significant interval where an MMN-like component is observed in the Fz electrode group. Topographies shown correspond to the P1 and MMN intervals respectively. Note that non-significant intervals and topographies are not shown. All topographies and waveforms are kept to the same scale for ease of comparison of amplitudes.

In addition to changes in the steady-state response, the appearance of a salient event in the auditory scene evoked changes in the ERPs. ERP components before and after the salient change in the stream were contrasted using pairwise *T*-tests. An analysis based on averaging 1-note immediately before and after the salient change (see Materials and Methods) revealed a significant change of the early negativity (possibly a long-latency N1 component) in the Cz central electrodes in the attend experiments (Figure 4, left column). A pairwise *T*-test revealed salient changes increased the amplitude of N1 significantly [*t*_(35)_ = −6.058, *p* < 0.001; Bonferroni adjustment for the following three comparisons *p* = 0.017: one-stream attend: *t*_(11)_ = −6.816, *p* < 0.001; two-stream attend: *t*_(11)_ = −4.765, *p* < 0.001; two-stream ignore: *p* = 0.426]. This component could conceal a mismatch negativity (MMN) component but cannot be clearly labeled as such because of its localization at central regions of the scalp especially in the attend experiments (Figure 4, left column). In addition, the analysis revealed a significant late P300 positive component in the attend conditions with a predominantly central distribution (Figure 4, middle column, Cz electrode group). A pairwise *T*-test confirmed the significant P300 peak [*t*_(35)_ = 5.074, *p* < 0.001; Bonferroni adjustment for the following three comparisons *p* = 0.017: one-stream attend: *t*_(11)_ = 2.830, *p* < 0.016; two-stream attend: *t*_(11)_ = 3.978, *p* < 0.002; two-stream ignore experiment: *t*_(11)_ = 2.142, *p* = 0.055].

To reveal any concealed ERP components that were not clearly visible from the 1-note averaging, the same analysis was repeated by averaging data from more trials including three notes before and after the salient change (see Materials and Methods). The 3-note analysis confirmed results observed in the late P300 components, with significant enhancement across all three experiments (data not shown in the Figure). The 3-note analysis also revealed a significant difference in the P1 component in the central Cz electrodes. A pairwise *T*-test revealed significant saliency induced amplitude increases [*t*_(35)_ = 6.009, *p* < 0.001; Bonferroni adjustment for the following three comparisons *p* = 0.017: one-stream attend: *t*_(11)_ = 4.012, *p* < 0.002; two-stream attend: *t*_(11)_ = 4.136, *p* < 0.002; two-stream ignore: *t*_(11)_ = 2.578, *p* < 0.026]. The analysis also revealed a frontocentrally distributed MMN-like component in the difference deviant/standard curve in one-stream attend and two-stream ignore experiments in the Fz electrode group (Figure 4, right column). A pairwise *T*-test showed significant saliency effect in general [*t*_(35)_ = −4.025, *p* < 0.001; Bonferroni adjustment for the following three comparisons *p* = 0.017: one-stream attend: *t*_(11)_ = −2.663, *p* < 0.022; two-stream ignore: *t*_(11)_ = −3.049, *p* < 0.011; two-stream attend: *p* = 0.161].

### Top-down attention and saliency effects

In order to tease apart the contribution of the attentional state of participants and the presence of a salient change in an acoustic stream, we first compared the 4.7 Hz ASSR component during the acoustic scene (prior to any salient change) in the two-stream attend vs. two-stream ignore experiments. Differences between these neural responses directly reflected the modulatory-effect of task-dependent attention given that the acoustic stimulation in both cases was identical. In other words, listeners were presented with the same physical sound parameters (2 streams) while we directed their attentional focus toward or away from the music stimuli. In line with previous findings in the literature (Elhilali et al., 2009; Xiang et al., 2010), the attended auditory stream revealed a significant increase in the stimulus phase-locked response with attention relative to the ignore experiment [*F*_(1, 22)_ = 6.115, *p* < 0.022, η^2^_*p*_ = 0.217] in the univariate analysis. This result confirms an effect of neural enhancement caused by top-down attention. Once the salient change occurred in the music note stream, both conditions showed significant additional increase in ASSR energy but with no significant interaction between the two experiments and the increase (*p* = 0.298) in the Two-Way ANOVA test. The lack of *differential* change in the phase-locked response suggests that the bottom-up saliency caused by the notable change introduced ASSR energy increase that was independent in the stream notes of the top-down attentional state (Figure 3B). This result further corroborates our earlier finding that bottom-up attention induced by salient changes in an auditory stream is likely reflected in enhanced phase-locking of neural responses entrained by the stimulation rate, independent of the top-down attentional state of listeners.

We further examined the lateralization of ASSR of the auditory stream and the change of ASSR due to the salient change of the acoustic scene by averaging all left electrodes and right electrodes in the two-stream attend and ignore experiments. Prior to the salient change, there was a significant left lateralization of the ASSR response in the two-stream attend experiment [pairwise *T*-test; *t*_(11)_ = 2.323, *p* < 0.040], but no significant lateralization in the two-stream ignore experiment (*p* = 0.140). This result is consistent with previously reported left-hemisphere biases during selective attention in auditory and visual tasks (Coch et al., 2005; Bidet-Caulet et al., 2007). The change in ASSR amplitude before and after the salient change did not show any significant lateralization effects under both of the two-stream attend (*p* = 0.141) and two-stream ignore (*p* = 0.310) experiments suggesting a lack of lateralization during bottom-up attention.

We also examined the ERP components of the acoustic scene prior to a salient change under different attentional states, in the two-stream attend and two-stream ignore experiments. Consistent with the previous analysis, the N1 components were derived from 1-note analysis and P1 components were derived from 3-note analysis. Prior to any salient change in the musical note stream, the MANOVA test with attend vs. ignore conditions as fixed factor and amplitudes of the two ERP components as dependent variables revealed a significant difference in P1 amplitude [*F*_(1, 22)_ = 8.578, *p* < 0.008, η^2^_*p*_ = 0.281], though no differences were observed in the N1 amplitude (*p* = 0.198). Once a salient change occurred in the stream, the Two-Way ANOVA with salient change and attend vs. ignore conditions as two factors on the four ERP components revealed further significant increase denoted by significant interactions between saliency and top-down attention in the P1 amplitude [*F*_(1, 22)_ = 5.308, *p* < 0.031, η^2^_*p*_ = 0.194], the N1 peak amplitude [*F*_(1, 22)_ = 12.401, *p* < 0.002, η^2^_*p*_ = 0.360] and the P300 component [*F*_(1, 22)_ = 5.472, *p* < 0.029, η^2^_*p*_ = 0.199]. No significant interaction were noted in the MMN-like component (*p* = 0.528). *Post-hoc T*-test comparisons of the amplitude changes from auditory stream to salient events under the attend and ignore conditions confirmed an enhanced saliency effect under top-down attention in the three aforementioned ERP components [P1: *t*_(22)_ = 2.304, *p* < 0.031; N1: *t*_(22)_ = −3.521, *p* < 0.002; P300: *t*_(22)_ = 2.339, *p* < 0.029; MMN-like: *t*_(22)_ = 0.641, *p* = 0.528]. This differential change between attend and ignore conditions hints to enhancement of bottom-up effects due to acoustic saliency that are mediated by top-down attention.

### The acoustic scene and saliency effects

Next, we explored the effect of the acoustic scene itself on the neural responses to the music notes, by comparing the one-stream attend and two-stream attend experiments. In both conditions, participants were attending to the same auditory stream and performing a similar task of salient change detection, but in presence (or absence) of a competing background stream. Attending to an auditory stream caused similar ASSR phase-locked responses, irrespective of whether the background contained a competing stream or not. The ASSR response was similar under both one-stream attend and two-stream attend conditions during the auditory stream (*p* = 0.916) in the univariate analysis. The introduction of a deviant change in the attended stream caused similar increases in the ASSR power under both one-stream and two-stream conditions (*p* = 0.171) shown in the Two-Way ANOVA test. The change in ASSR energy appeared to be marginally left lateralized in the one-stream attend experiment [*t*_(11)_ = 1.805, *p* = 0.098], but not in the two-stream attend experiment (*p* = 0.141). There was no significant difference between listening to one-stream and two-stream conditions in the N1 and P1 ERP responses (Figure 4) during the auditory stream in the MANOVA test. No significant interactions between saliency and scene complexity were found in the Two-Way ANOVA test on all four ERP components.

## Discussion

The current study focuses on how bottom-up, stimulus-driven salient changes in a dynamic scene modulate the neural representation of an auditory stream under different states of top-down attentional focus and with different complexities of the acoustic scene. Specifically, we report that a salient change evokes an increase in the phase-locked steady-state response to this salient event, irrespective of whether subjects attended to it or not. Given the complex nature of saliency changes in the current paradigm (across different acoustic dimensions defined along non-deterministic distributions), the observed ASSR changes likely reflect neural generators operating across larger neuronal groups or different neural centers (Escera et al., 2014); rather than mechanisms operating at the single neuron level such as stimulus-specific adaptation (SSA) which modulates the neural response to rare, unexpected or prominent events but is gated by the tuning properties of each neuron (Nelken, 2014). There was an observed left lateralization once attention is directed to the auditory stream (relative to the ignore condition), consistent with previous reports of a functional role of the left hemisphere in selective attention (Zani and Proverbio, 1995; Coch et al., 2005; Bidet-Caulet et al., 2007). An intriguing observation is the time-locked effect caused by the salient change response rather than a global, broad entrainment reflecting more general, non-specific enhancement in the neural response as previously reported with top-down attention (Picton and Hillyard, 1974a; Hari et al., 1980). Such specificity hints to a closer role of sensory cortex in coding the salience and dynamics of acoustic events with high degree of fidelity. The ASSR increase due to salient change in the scene appears to supplement further ASSR increase induced by top-down attention; which could itself be mediated by adaptive changes in the response characteristics of cortical neurons via mechanisms of neuronal plasticity (Fritz et al., 2007b). The current report of ASSR modulation with saliency of acoustic event is in agreement with earlier studies (Elhilali et al., 2009), but goes further in arguing for independent mechanisms underlying bottom-up induced ASSR changes and top-down attentional state. This bottom-up/top-down dissociation is further supported by the comparable size of ASSR increase which builds on top of increases boosted by purely top-down effects. Similar observations were made in the visual literature; whereby an increase in the steady-state visual evoked potential (SSVEP) is evoked by top-down attention; while the degree of change caused by different levels of saliency are generally the same under attend and non-attend states (Andersen et al., 2012).

An earlier report of changes in the ASSR energy due to salient event noted an opposite effect to the one reported here. A study by Rockstroh et al. (1996) found a reduction of ASSR power at 40 Hz after a deviant in a typical oddball paradigm, accompanied by a P300 component. A plausible explanation for the discrepancy between these two reports is that our definition of an auditory stream was characterized within tempi directly matched to time constants where auditory streaming is often observed, of the order of a few Hertz (Moore and Gockel, 2002). These are believed to have direct biophysical underpinnings matched to response dynamics of cortical neurons, whose selectivity to temporal rates is also of the order of a few Hertz (Miller et al., 2002; Liégeois-Chauvel et al., 2004). Rockstroh et al. (1996) examined deviance in a range where no reported cortical phase-locking exists, and is likely evoking different mechanisms than those involved in auditory stream formation and tracking.

An interesting aspect of the experimental paradigm used in the current study is that it did not follow a classic oddball paradigm, and was not defined by any repetitive tokens or specific sequence. Rather, the musical note stream of interest consisted of a series of complex tones (synthesized music instrument sound) with varying acoustic parameters randomly following a restricted distribution along a given acoustic dimension, e.g., timbre, pitch, duration, or intensity. The auditory tokens used were dynamic but restricted to a range of parameters that likely groups them into a cohesive auditory stream. For instance, the pitch variations were confined within three semitone range, largely within natural contours expected in natural speech (Nooteboom, 1997) and also consistent with frequency variations tested in auditory streaming with varying frequency components (Bregman, 1990). Despite the fluctuating patterns in the stream, the ability of listeners to detect big deviations from its underlying statistical distribution strongly suggests that the auditory system is collecting statistics about the inherent variations in the stream, and either forming unconfined templates of the average distribution, representative statistics and parameters of the acoustic space or estimates of the underlying dynamical system of the stimulus that can be used to predict fluctuations in the scene (Sussman, 2007; D'Antona et al., 2013; Simpson et al., 2014).

Our analysis of event-related potentials reveals that salient change-induced ASSR changes are accompanied by attention-dependent modulation of the early P1, N1, and P300 components. Earlier reports corroborate an increase in the amplitude of the N1 component by involuntary attention (Näätänen et al., 1978). The observed change in both P1 and N1 peaks is likely reflecting the salient nature of the change introduced in the ongoing stream. It could mark the reset of the grouping process, a flag of aberrant events within the existing stream or initiation of a new auditory stream which does not fit within the expected fluctuations of the ongoing stream (Winkler et al., 2009). The differential effect of top-down attention on both P1 and N1 components with salient change implies a complex interaction of bottom-up deviance detection with top-down attention, though only the P1 component was modulated by purely top-down attention when comparing the attend and ignore cases prior to any salient change in the stream. Moreover, in agreement with most deviance detection studies, an MMN-like component was also observed with the salient change under attend and ignore conditions though not in the two-stream attend (Näätänen and Kreegipuu, 2012). This was accompanied by a late P300 component that was associated with discrimination in all three experiments (Martin et al., 1999; Martin and Stapells, 2005; Näätänen et al., 2007). It is important to note that dissociating the observed N1 and MMN-like component given the complexity of the task was non-trivial and mostly based on diverging scalp topographies for the two components. The observed mismatch negativity reported here was called “MMN-like” to guardedly reflect the peculiarity of this component in the current study. However, as previously noted by competing hypotheses of mismatch negativity generation, the distinction between MMN and N1 components always warrants caution and careful methodological strategies that are not easily attainable in the current design (Näätänen and Winkler, 1999; Garrido et al., 2009; May and Tiitinen, 2010; Lozano-Soldevilla et al., 2012).

Finally, the manifestation of a salient event was investigated as a function of the acoustic properties of the scene. A salient change occurring in an auditory stream that exists in isolation induces a notable MMN-like component that is not visible in presence of a competing stream under top-down attention, though both cases were associated with similar increases in the ASSR amplitude and involved comparable attentional loads as indicated by similar behavioral performances. The association between ASSR power increases and saliency is in agreement with effects reported earlier in both auditory and visual studies (Elhilali et al., 2009; Andersen et al., 2012), though earlier works have also observed a decrease of the P1 amplitude with delays of P1 latency (Billings et al., 2009) as well as other ERP components (Martin et al., 1999; Martin and Stapells, 2005; Lagemann et al., 2010) under a masking noise stream.

Overall, the current study reinforces the notion that auditory perception of complex acoustic scenes engages complex processes of statistical inference and dynamical tracking that define an auditory stream as an amorphous structure defined in a statistical sense. It is relative to this baseline that the system is able to flag any salient, deviant or unexpected events in a seemingly automatic, sensory-driven fashion. However, top-down attention reinforces the bottom-up saliency detection with mechanisms that are manifested mostly in amplitude of both early and late ERP components. Both forms of attention induce enhanced phase-locked responses to the ongoing rhythm of the auditory stream and may underlie the brain's ability to not only switch attention amongst simultaneous sounds streams, but also adjust its interpretation of the auditory stimulus depending on the acoustic parameters of the sensory input.

### Conflict of interest statement

The authors declare that the research was conducted in the absence of any commercial or financial relationships that could be construed as a potential conflict of interest.
